# Symptomatic Subsidence of a Box-Shaped Titanium Cage After Anterior Cervical Discectomy and Fusion: Two Case Reports and Review of the Literature

**DOI:** 10.7759/cureus.63933

**Published:** 2024-07-05

**Authors:** Satoshi Tanaka, Shinsuke Yoshida, Ryosuke Tomio, Norio Ichimasu, Ai Kawaguchi

**Affiliations:** 1 Department of Neurosurgery, Tamus Sakura Hospital Kawaguchi, Kawaguchi, JPN; 2 Department of Neurosurgery, Saitama Medical Center, Kawagoe, JPN; 3 Department of Neurosurgery, Honjo Neurosurgery and Spinal Surgery, Honjo, JPN; 4 Department of Neurosurgery, Tokyo Medical University Hospital, Tokyo, JPN; 5 Department of Neurosurgery, Teikyo University School of Medicine, Tokyo, JPN

**Keywords:** anterior cervical discectomy and fusion (acdf), subsidence, cervical disc herniation, titanium box cage, cervical spondylosis

## Abstract

This study reports two cases of rare symptomatic subsidence of titanium cages after anterior cervical discectomy and fusion (ACDF). First, an 82-year-old man underwent ACDF at C5/6 and C6/7 using two 6 mm height box-type titanium cages. On the 34th postoperative day, motor weakness occurred in the right upper limb, and CT showed that the cage at C5/6 had subsided 6 mm into the C6 vertebral body. On postoperative day 55, both cages were removed, and C6 corpectomy was performed. The C5-7 space was refixed with a mesh cage and plate. He was discharged home from the rehabilitation hospital three months later. Second, a 41-year-old man underwent ACDF at C5/6 and C6/7 using two 5 mm height box-type titanium cages. He fell violently on the 33rd postoperative day, causing pain from the neck to the left hand, weakness, and skillful movement disorder in the left hand, and CT showed that the cages at C5/6 and C6/7 had subsided by 7 mm and 6 mm, respectively. On the 65th postoperative day, both cages were removed by reoperation, and C6 and 7 corpectomy was performed. The space between C5 and T1 was refixed with a mesh cage and plate. He was discharged home two months later. Possible causes of titanium cage subsidence include osteoporosis, trauma, vertebral cortex damage by an operative procedure, and cage height of 6 mm or more. While ACDF is safe and effective for cervical spondylosis, special caution is needed in older osteoporotic patients.

## Introduction

Anterior cervical discectomy and fusion (ACDF) is widely used for cervical spondylotic myelopathy and radiculopathy, central cervical cord injury, and cervical disc herniation [1]. Laminoplasty is often performed for the ossification of the posterior longitudinal ligament (OPLL) of the cervical spine and spinal compressive lesions of three or more intervertebral spaces [2]. Laminoplasty has many complications, such as axial pain, kyphotic deformity, and C5 palsy, and ACDF is preferred especially for spinal compressive lesions of two or fewer vertebrae [3]. In ACDF surgery, titanium cages have been used for a long time instead of autologous iliac bone grafts. As a complication of ACDF, hoarseness and dysphagia are known to occur regardless of the presence or absence of recurrent laryngeal nerve palsy. Serious complications, such as respiratory problems and esophageal injuries, are extremely rare in ACDF by neurosurgical microsurgery. Although the subsidence of cages used as implants has been reported to occur relatively frequently, symptomatic cage subsidence was relatively rare [4]. The authors examined two cases of symptomatic subsidence of box-shaped titanium cages.

## Case presentation

Case 1

**Figure 1:**
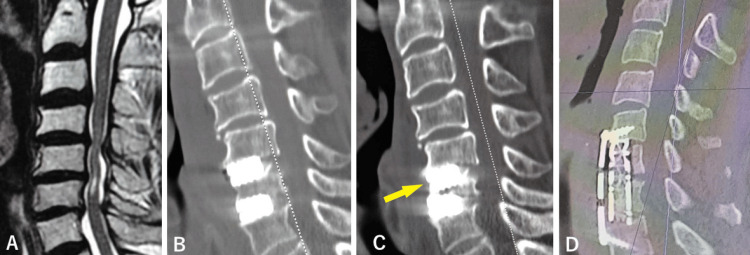
Case 1: Sagittal MRI and CT scan before and after two operations. T2-weighted MRI sagittal image before the first operation (A), sagittal reconstructed CT scans after the first operation showing that sufficient decompression was achieved (B), 37 days after the operation showing  that the cage at C5/6 had subsided 8 mm to the C6 vertebral body (C, arrow), after the second operation showing the proper position of the mesh and plate and good cervical spine alignment (D). MRI: magnetic resonance imaging

An 82-year-old male had suffered from motor weakness and paresthesia in his right hand for the previous six months and walked with a rollator. He was admitted a month before the operation when he became unable to walk. Neurological findings included right-hand motor weakness, skillful movement disorder, and gait disturbance. Cervical spine magnetic resonance imaging (MRI) showed spinal canal and foraminal stenosis at the right C5/6 and 6/7 levels. Intramedullary T2 high signal intensity was seen at the C5/6 level (Figure 1A). The preoperative Japan Orthopedic Association (JOA) score was 5.5/17 [5].

**Figure 2:**
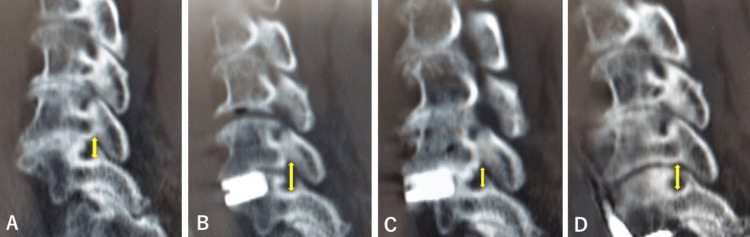
Case 1: Sagittal reconstructed CT scan. Sagittal reconstructed CT scan showing that the diameter of the right C5/6 intervertebral foramen (⇕) was 7.0 mm before the first operation (A), 11.0 mm after the first operation (B), 4.0 mm before the second operation (C), and 9.2 mm after the second operation (D).

C5/6 and C6/7 ACDF with titanium box cages was performed from the right side. Two 6-mm height m-cage SR (Ammtec, Tokyo, Japan) were inserted. The postoperative JOA score was 7.5/17 on the day after the operation. Marked recovery from right-hand motor weakness was observed, and gait was possible with a rollator a week after the operation. Postoperative computed tomography (CT) scans a day after the operation showed that sufficient decompression was achieved (Figure 1B). He was transferred to a rehabilitation hospital 28 days after the operation. Six days after admission to the rehabilitation hospital, he experienced dizziness and motor weakness in the right extremities. He was readmitted to our hospital nine days after discharge. A cervical CT scan showed that the cage at C5/6 had subsided 8 mm to the C6 vertebral body (Figure 1C), and compression of the right C6 root due to stenosis of the right C5/6 intervertebral foramen seemed to be the cause of right-hand motor weakness (Figure 2A-2C).

The second operation 55 days after the first operation consisted of C6 corpectomy and ACDF with a mesh-cage filled with autologous bone graft and hydroxy apatite ceramic pieces and a plate fixed with four mini-screws. Intraoperative motor-evoked potential (MEP) monitoring showed no decrease in amplitude. The postoperative JOA score recovered from 6 to 8. The postoperative CT scan the day after the second operation showed that the mesh and plate were in the proper position and had good cervical spine alignment (Figure 1D). The diameter of the right C5/6 intervertebral foramen was sufficiently recovered with neurological improvement (Figure 2D). He was again transferred to the rehabilitation hospital and walked out of the hospital three months later.

Case 2

**Figure 3:**
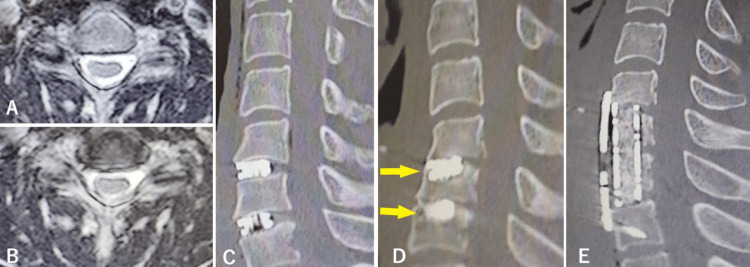
Case 2: Sagittal MRI and CT scan before and after two operations. T2-weighted MRI axial image before the first operation at C5/6 (A) and C6/7 (B), sagittal reconstructed CT scans after the first operation showing that sufficient decompression was achieved (C), 37 days after the operation showing that the cage at C5/6 had subsided by 7 mm and the cage at C6/7 had subsided by 6 mm (D, arrows), and after the second operation showing the proper position of the mesh and plate and good cervical spine alignment (E). MRI: magnetic resonance imaging

A 41-year-old man was rear-ended while driving a car, suffered a fractured right rib and right hemiparesis, and underwent conservative treatment. In the sixth month after the injury, he was urgently hospitalized due to weakness in the upper right limb and skillful movement disorder during abdominal exercises. Neurological findings included right-hand motor weakness and skillful movement disorder. The grasping force of his right hand was 23.2 kg. MRI revealed C5/6, 6/7 right foraminal stenosis (Figure 3AB). The preoperative JOA score was 12.5.

**Figure 4:**
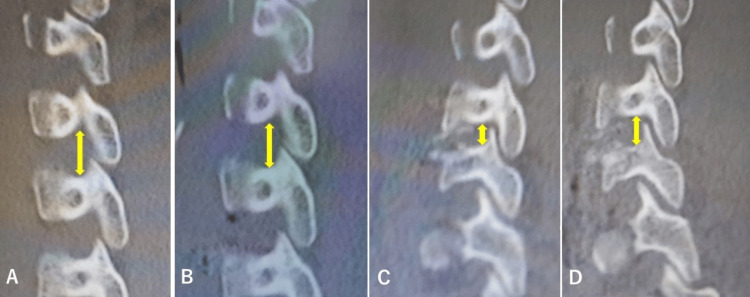
Case 2: Sagittal reconstructed CT scan. Sagittal reconstructed CT scan showing that the diameter of the left C5/6 intervertebral foramen (⇕) was 9.0 mm before the first operation (A), 9.3 mm after the first operation (B), 4.9 mm before the second operation (C), and 7.9 mm after the second operation (D).

Seven months after the injury, ACDF at the C5/6 and C6/7 intervertebral spaces was performed from the left side using two 5-mm height m-cage SR. His JOA score recovered to 16.5 postoperatively. The grasping force of his right hand was restored to 35.5 kg. Immediate postoperative CT scan showed that sufficient decompression was achieved (Figure 3C). Although he was discharged from the hospital and returned to work, he fell violently on the 33rd postoperative day, causing pain from the neck to the left hand, weakness (grasping force 5.6 kg), and skillful movement disorder in the left hand. His CT showed that the cage at C5/6 had subsided by 7 mm and the cage at C6/7 had subsided by 6 mm (Figure 3D). Compression of the left C6 root due to stenosis of the right C5/6 intervertebral foramen seemed to be the cause of right-hand motor weakness (Figure 4A-4C).

On the 65th postoperative day, the two cages were removed by reoperation and C6 and 7 corpectomy was performed; the space between C5 and T1 was refixed with a mesh cage filled with autologous bone graft and a plate fixed with four mini-screws. His JOA score recovered from 12.5 to 16 postoperatively, and the grasping force of his left hand was restored to 8.5 kg. A postoperative CT scan on the day after the second operation showed that the mesh and plate were in the proper position and had good cervical spine alignment (Figure 3E). The enlargement of the sagittal diameter of the left C5/6 intervertebral foramen is shown in Figure 4D. He was discharged home three months after the second operation.

## Discussion

Currently, cervical spinal surgery is divided into ACDF and laminectomy or laminoplasty via a posterior route. Whether anterior or posterior surgery is performed is determined according to the number of affected intervertebral spaces, the presence of spinal canal stenosis, myelopathy, or radiculopathy and the proficiency of the surgeon. Cage subsidence was usually defined as significant at 3 mm or more [6]. The subsidence of the titanium cage varied greatly from 4% to 56% [7,8]. Patients who underwent reoperation were considered to have had symptomatic subsidence. The rate of revision surgery was from 0% to 13%, and the symptomatic subsidence of titanium cages after ACDF was extremely rare [4,7,8].

The results of the literature seemed to suggest that ACDF should be preferred over laminoplasty for the treatment of lesions of three intervertebral spaces or less since ACDF was associated with less intraoperative blood loss and better preservation of cervical lordosis [3]. Multilevel laminoplasty showed better radiological and similar clinical outcomes compared to three-level ACDF, which has more surgical complications [9]. An anterior approach is believed to be safe and effective, at least for two vertebral levels, because a posterior approach is associated with several complications such as kyphotic deformity due to the destruction of proper cervical spinal alignment, axial nuchal pain due to destruction of posterior supporting elements, and C5 palsy due to extension of a nerve root by excessive dilatation of the spinal canal [3,10]. These complications occur quite frequently. On the other hand, ACDF rarely shows complications except for hoarseness, dysphagia due to or not due to recurrent laryngeal nerve palsy, and Hornell syndrome due to effects on sympathetic nerves [3,4,11]. These complications are almost always mild and reversible. Postoperative newly developed motor palsy with intramedullary high signal intensity on T2-weighted image of cervical MRI, so-called white cord syndrome, has been reported to occur three times more frequently in laminoplasty than in ACDF [12]. The prevalence of an unsatisfactory outcome where the patient did not recover to the preoperative level by a six-month follow-up after cervical laminoplasty for the ossification of the posterior longitudinal ligament in a multi-institutional retrospective study was 7/581 (1.2%) [13]. Devastating complications of spinal decompression surgery such as C5 palsy and white cord syndrome have rarely been reported with ACDF [12,14].

ACDF was initially performed using autologous iliac bone fragments as intervertebral spacers [1]. Postoperative pain in the iliac fragment collection area became a problem, and intervertebral cages were used for ACDF [15]. There have been reports that the subsidence rate was similar between cages and autologous bones, but there are still many reports of cage subduction in the literature [4,7,8,16].

Subsidence of a box cage, which is thought to be less common than that of a cylinder cage, has been reported [17]. In the cylinder-cage group, the average postoperative restoration of disc height was 72%, but with a narrowing rate of 26% in follow-up. In the carbon box group, postoperative disc height restoration was 51% with narrowing in follow-up to 6%. Although subsidence of the cylinder cage has been recorded at a long postoperative follow-up, symptomatic subsidence seems to be rare. On the other hand, the symptomatic subsidence of the titanium box cage that we experienced in this study occurred as early as about one month after surgery in both cases. Subsidence due to long-term follow-up of the cylinder-type cage was considered to be less likely to cause symptoms, and it was unlikely to be a clinical problem. Subsidence of a cylinder cage developed gradually, and compression of the nerve root by acute narrowing of the intervertebral foramen as shown in the present cases was rare. Subsidence of the cage was not necessarily accompanied by stenosis of the intervertebral foramen and newly developed neurological disorders.

Polyetheretherketone (PEEK) cages have often been reported to have less subsidence than titanium cages [6,18]. However, it was also reported that there were no significant differences in clinical or radiological outcomes between titanium and PEEK cages [6]. Subsidence was also observed in cases where a plate was used in combination with a cage, but cases that require revision surgery were extremely rare [19]. Again, radiological cage subsidence was less related to clinical outcomes [4,7,8].

Subsidence of the box cage in Case 1 seemed to have been due to osteoporosis because of aging, destruction of the vertebral cortex during the widening of the intervertebral space by a Casper spreader, and the height of the cage: as the cage height increases, the risk of cage subsidence in ACDF increases. A PEEK cage sinks more frequently with a height of 5.5 mm or more, although the authors usually used a cage 6 mm high [18]. More recently, we have used 5 mm-height titanium box cages. Currently, if the cortex was damaged by an intraoperative Casper destructor or the like, it was repaired with bone cement. Preoperative bone densitometry was performed, more than 80% of patients without osteoporosis were given postoperative soft collars for two weeks, and less than 80% of osteoporotic patients wore collars postoperatively for one month.

Recently, artificial cervical discs have also been used without fixation in anterior cervical decompression [20]. The mobility of the cervical spine is maintained by using artificial cervical discs. However, the installation of artificial discs requires extensive decompression up to both Luschka joints, and operative invasion is greater than in ACDF. In addition, postoperative pain may occur compared to the case where it is fixed in a cage. Artificial cervical disc replacement may not be a replacement for ACDF.

## Conclusions

We have reported two cases of rare symptomatic subsidence of titanium cages after ACDF. Possible causes of titanium cage subsidence include osteoporosis, vertebral cortex damage by an operative procedure, and cage height of 6 mm or more. Currently, bone densitometry is measured before ACDF surgery, and soft collars are worn for one month after surgery for patients with osteoporosis and two weeks for those without osteoporosis.
